# In vivo biocompatibility evaluation of in situ-forming polyethylene glycol-collagen hydrogels in corneal defects

**DOI:** 10.1038/s41598-021-03270-3

**Published:** 2021-12-13

**Authors:** Yoon Hong Chun, Sun-Kyoung Park, Eun Jeong Kim, Hyun Jong Lee, Hyewon Kim, Won-Gun Koh, Gabriella Fernandes Cunha, David Myung, Kyung-Sun Na

**Affiliations:** 1 Department of Pediatrics, Incheon St. Mary’s Hospital, College of Medicine, The Catholic University of Korea, Incheon, 21431 Republic of Korea; 2 Department of Ophthalmology, Yeouido St. Mary’s Hospital, College of Medicine, The Catholic University of Korea, 10, 63-ro, Yeongdeungpo-gu, Seoul, 07345 Republic of Korea; 3 Chemical and Biological Engineering, Gachon University, Seongnam‐si, Gyeonggi‐do 13120 Republic of Korea; 4 Department of Chemical and Biomolecular Engineering, Yonsei University, Seodaemun-gu, Seoul, 03722 Republic of Korea; 5 Ophthalmology, Byers Eye Institute at Stanford University School of Medicine, Palo Alto, CA 94303 USA

**Keywords:** Drug development, Corneal diseases

## Abstract

The available treatment options include corneal transplantation for significant corneal defects and opacity. However, shortage of donor corneas and safety issues in performing corneal transplantation are the main limitations. Accordingly, we adopted the injectable in situ-forming hydrogels of collagen type I crosslinked via multifunctional polyethylene glycol (PEG)-N-hydroxysuccinimide (NHS) for treatment and evaluated in vivo biocompatibility. The New Zealand White rabbits (N = 20) were randomly grouped into the keratectomy-only and keratectomy with PEG-collagen hydrogel-treated groups. Samples were processed for immunohistochemical evaluation. In both clinical and histologic observations, epithelial cells were able to migrate and form multilayers over the PEG-collagen hydrogels at the site of the corneal stromal defect. There was no evidence of inflammatory or immunological reactions or increased IOP for PEG-collagen hydrogel-treated corneas during the four weeks of observation. Immunohistochemistry revealed the presence of α-smooth muscle actin (α-SMA) in the superior corneal stroma of the keratectomy-only group (indicative of fibrotic healing), whereas low stromal α-SMA expression was detected in the keratectomy with PEG-collagen hydrogel-treated group. Taken together, we suggest that PEG-collagen may be used as a safe and effective alternative in treating corneal defect in clinical setting.

## Introduction

Approximately 36 million individuals are diagnosed with blindness annually worldwide, and corneal opacity is considered a major cause of blindness^1^. Corneal transparency is lost after corneal trauma, burns, and various inherited or acquired diseases^2^. Current therapeutic options for corneal stromal opacity or scarring are limited to corneal transplantation^3^. When the corneal epithelium and stroma are affected, anterior lamellar keratoplasty can be selected for partial corneal transplantation, in which only the stromal part of the cornea is replaced, and the unaffected healthy corneal endothelium is retained^4^. The supply of donor cornea is insufficient, especially in Asia, due to cultural barrier or lack of information^5^. Several synthetic alternatives to a native tissue have been proposed for corneal stromal replacement^3,6–8^. The prerequisites of an engineered tissue to be used as corneal stromal substitutes are biocompatibility to overcome the immune reaction, transparency for vision, and strength to sustain external and internal pressure^3,9^.

**Figure 1:**
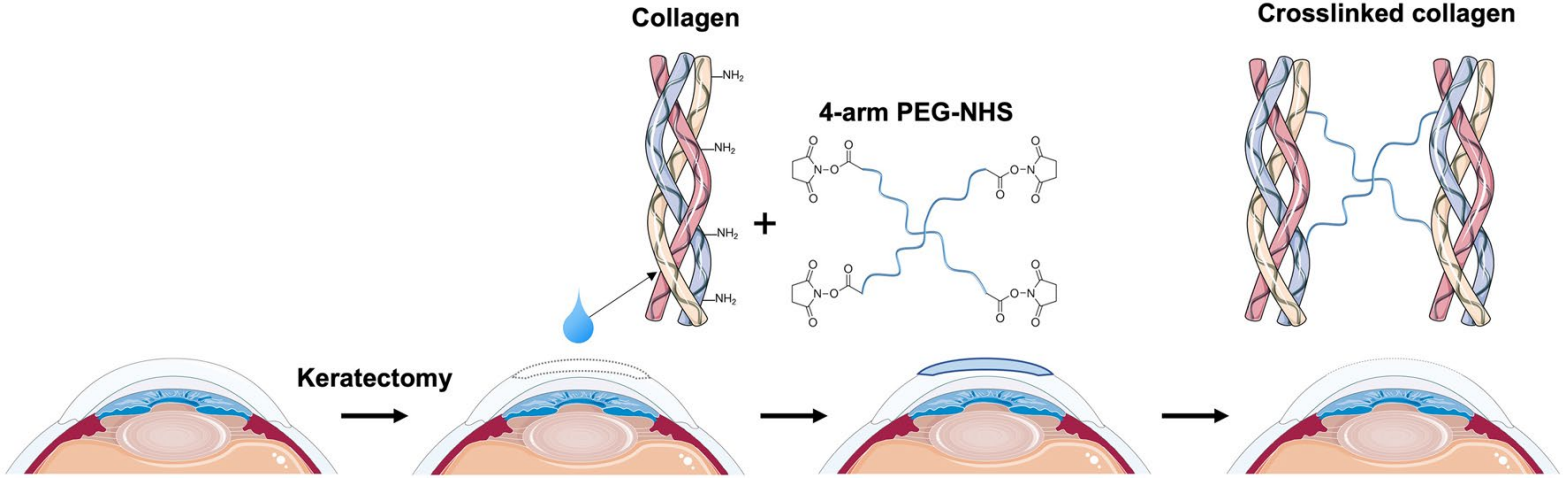
Schematic diagram showing the injection of the in situ-forming polyethylene glycol-collagen hydrogel into an in vivo rabbit corneal defect model. Parts of the figure were drawn by using pictures from Servier Medical Art (http://smart.servier.com/), licensed under a Creative Commons Attribution 3.0 Unported License (https://creativecommons.org/licenses/by/3.0/).

Type I collagen is the most well-established natural polymer, which is the most abundant in the human body^10^. It is also the major component of extracellular matrix (ECM) in the corneal stroma, where it exists as highly regular fibrils for structural support and transparency^11^. The fibrillary structure is stabilized by posttranslational modification that allows the formation of intermolecular and interfibrillar crosslinks^12,13^. Thus, the crosslinked collagen has been used to prepare scaffolds for tissue repair and engineering by increasing stability and control degradation^3,8,10^. Moreover, combining synthetic polymer with natural polymer collagen has been widely studied to increase strength and control the degradation rate^14–16^. In a previous study, Samarawickrama et al. prepared collagen-like peptide conjugated to PEG-maleimide to seal acute corneal perforations while promoting tissue regeneration^17^. Furthermore, Fernandes-Cunha et al. have reported the development of an in situ-forming hydrogel of collagen type I crosslinked via multifunctional polyethylene glycol (PEG)-N-hydroxysuccinimide (NHS) and characterized its biophysical properties and regenerative capacity^18^. In the present study, we aimed to evaluate the in vivo biocompatibility of in situ-forming PEG-collagen hydrogels in corneal defects of rabbits for 4 weeks (Fig. 1).

## Results

### Mechanical and optical properties of PEG-collagen hydrogels

**Figure 2:**
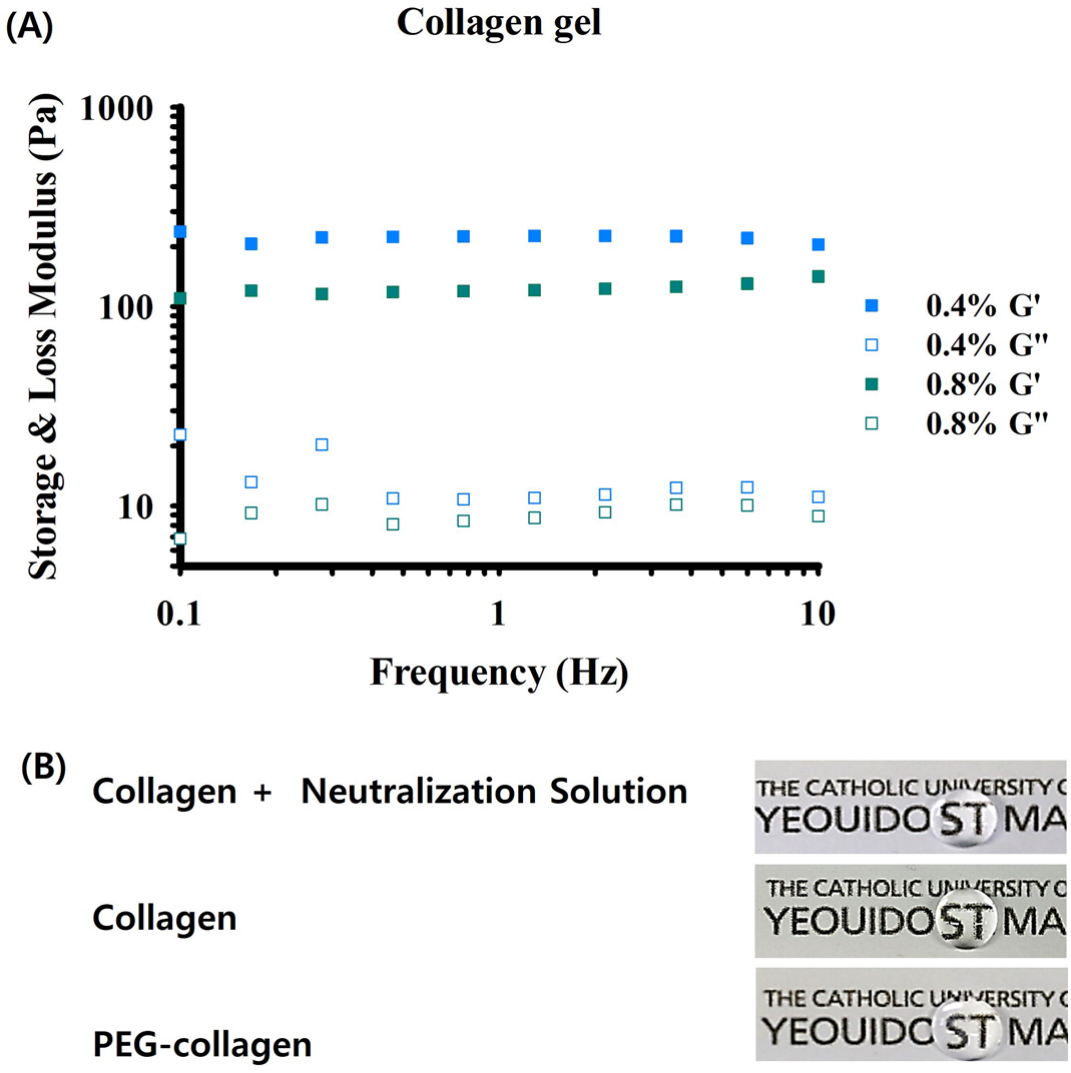
(**A**) Dynamic moduli of 0.4% and 0.8% 4-arm polyethylene glycol (PEG)-collagen as a function of frequency. (**B**) Photograph showing the clarity of 0.8% 4-arm PEG-collagen, non-crosslinked collagen, and collagen with neutralization solution. Both crosslinked and non-crosslinked (physical) collagens showed fair transparency.

The PEG-collagen hydrogels fabricated by mixing neutralized collagen and 4-arm PEG-NHS showed higher storage modulus than loss modulus for both 0.4 and 0.8% concentrations. The storage and loss moduli did not vary as a function of the frequency, and we confirmed the completion of gelation (Fig. 2A). The storage modulus of 0.4 and 0.8% PEG-collagen hydrogels were 221 ± 9 and 123 ± 8 Pa, respectively. The storage modulus of 0.4% PEG-collagen hydrogel was higher than 0.8% PEG-collagen hydrogel. In this study, we chose the 0.8% concentration of 4-arm PEG-NHS for followed experiments. The optical properties of PEG-collagen and non‐crosslinked collagen hydrogels were analyzed to determine if the crosslinked hydrogels were suitable for use in the cornea. The chemically crosslinked PEG-collagen hydrogel was shown to be transparent which was comparable with the non‐crosslinked collagen hydrogel (Fig. 2B).

### Clinical examinations

**Figure 3:**
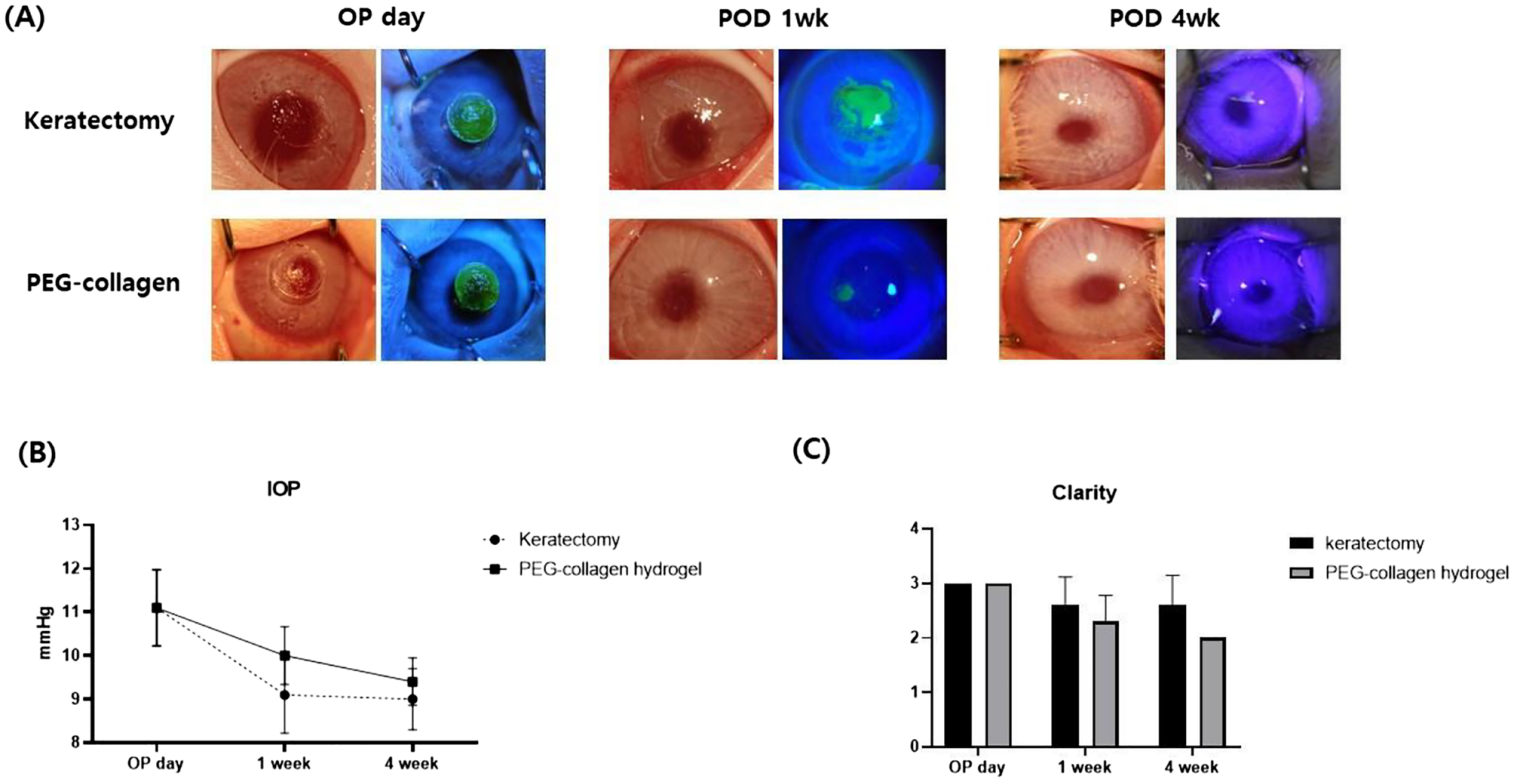
Representative anterior photographs (**A**), intraocular pressure (**B**), and corneal clarity grading by slit-lamp examination (**C**) of an in vivo rabbit keratectomy model without treatment and with polyethylene glycol-collagen hydrogel application immediately after keratectomy as well as 1 week and 4 weeks after surgery.

There were no signs of abnormalities on the ocular surface before surgery. Following anterior keratectomy, a round corneal defect, 5 mm in diameter, was detected at the center of the cornea, which was stained after fluorescein dyeing with blue light. All corneas subjected to keratectomy lost their transparency moderately. After 1 week, the corneal defect in the keratectomy-only group remained as evidenced by fluorescence staining. However, the keratectomy with 0.8% 4-arm PEG-collagen-hydrogel-treated group showed faster wound healing in the defect site than the keratectomy-only group (Fig. 3A). All corneas treated with PEG-collagen hydrogel showed no evidence of inflammation, such as conjunctival infection, stromal infiltration, chamber flare, keratic precipitation, and corneal neovascularization, over 1–4 weeks of follow-up. IOP in the keratectomy-only group was 11.1 ± 0.9, 9.0 ± 0.8, and 9.0 ± 0.7 mmHg on the day of surgery, 1 week after surgery, and 4 weeks after surgery, respectively. IOP in the keratectomy with PEG-collagen hydrogel-treated group was 11.1 ± 0.8, 10.0 ± 0.7, and 9.4 ± 0.5 mmHg on the day of surgery, 1 week after surgery, and 4 weeks after surgery, respectively (Fig. 3B). Statistical analysis showed no differences between the groups at each time point (*p* = 0.56, p = 0.60, and *p* = 0.22, respectively). The corneal opacity grades at 1 week and 4 weeks following surgery were 2.6 ± 0.5 and 2.6 ± 0.5 in the keratectomy-only group and 2.3 ± 0.5 and 2.0 ± 0.7 in the keratectomy with PEG-collagen hydrogel-treated group, respectively (Fig. 3C). Corneal haze was observed in both keratectomy-only and keratectomy with PEG-collagen hydrogel-treated groups, with no significant differences at all time points (*p* = 0.13 and *p* = 0.22, respectively).

**Figure 4:**
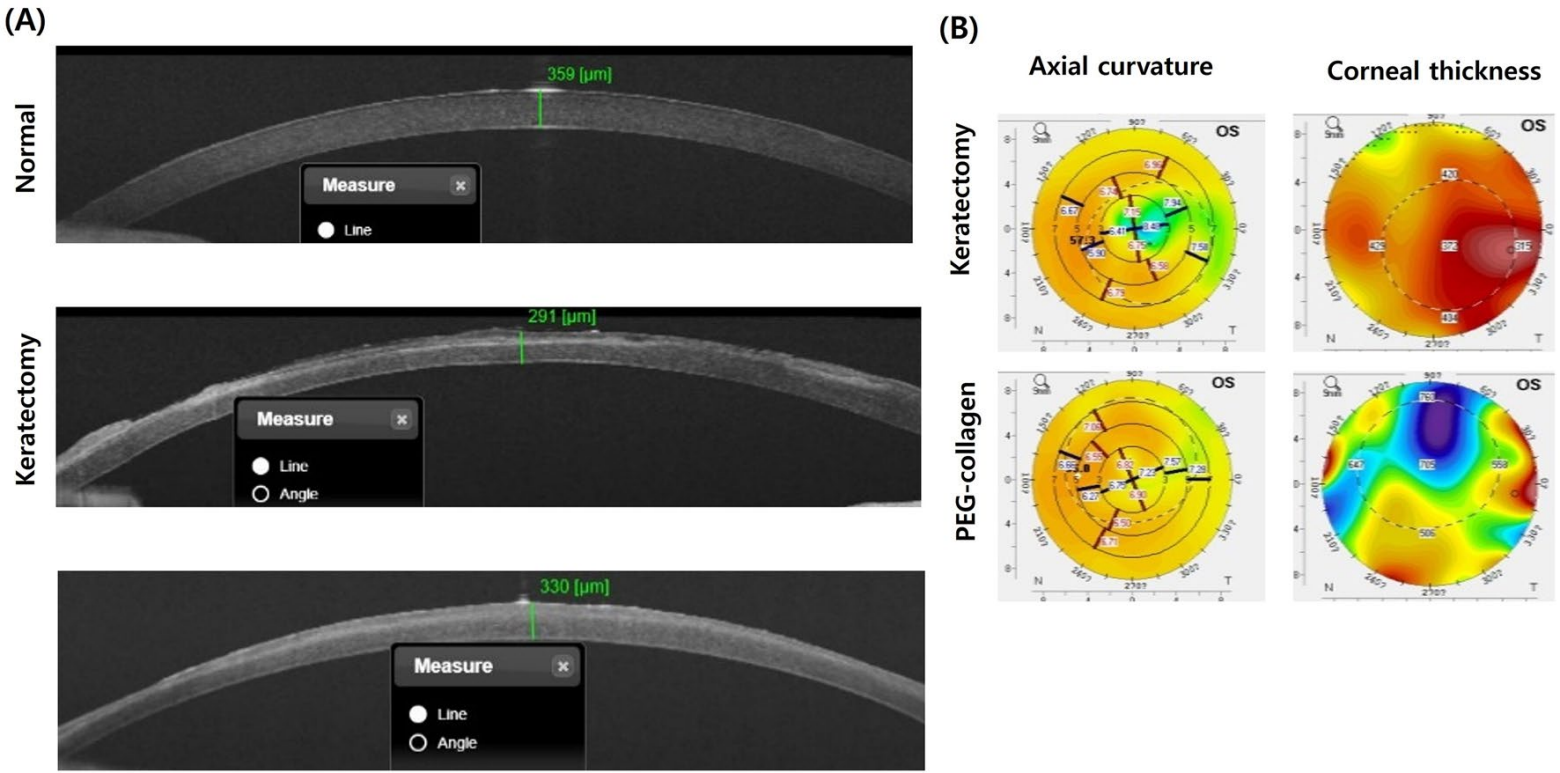
(**A**) Anterior segment optical coherence tomography (AS-OCT) cross-sectional images on the horizontal meridian at the center of the cornea, and (**B**) corneal imaging of axial curvature and corneal thickness measured using a rotating Scheimpflug camera after 4 weeks of surgery in the keratectomy-only and keratectomy with polyethylene glycol (PEG)-collagen hydrogel-treated group. AS-OCT revealed higher density (arrow), suggesting scarring at the center of the epithelial layer in the untreated keratectomy-subjected cornea than the keratectomy-subjected cornea treated with PEG-collagen, which showed high focal density area at the paracenter of the cornea. The regular anterior corneal surface and regular corneal thickness were increased in the keratectomy with PEG-collagen hydrogel-treated group compared with the keratectomy-only group.

Results of AS-OCT of the normal cornea revealed the outermost tear film as a high-intensity thin layer of the cornea, and the low-intensity thin layer beneath it represented the epithelium. A demarcation line between keratectomy and corneal scarring was observed, which appeared as areas of stromal hyperreflectivity that were more prominent in the keratectomy-only group than in the keratectomy with PEG-collagen hydrogel-treated group. Corneal thickness was 301.3 ± 15.8 μm in the keratectomy-only group and 320.4 ± 22.1 μm in the PEG-collagen hydrogel-treated group and normal corneas, however, there was no statistical differences (Fig. 4A). Pentacam images of both groups showed irregular astigmatism on the corneal surface (Fig. 4B).

### Histological findings

**Figure 5:**
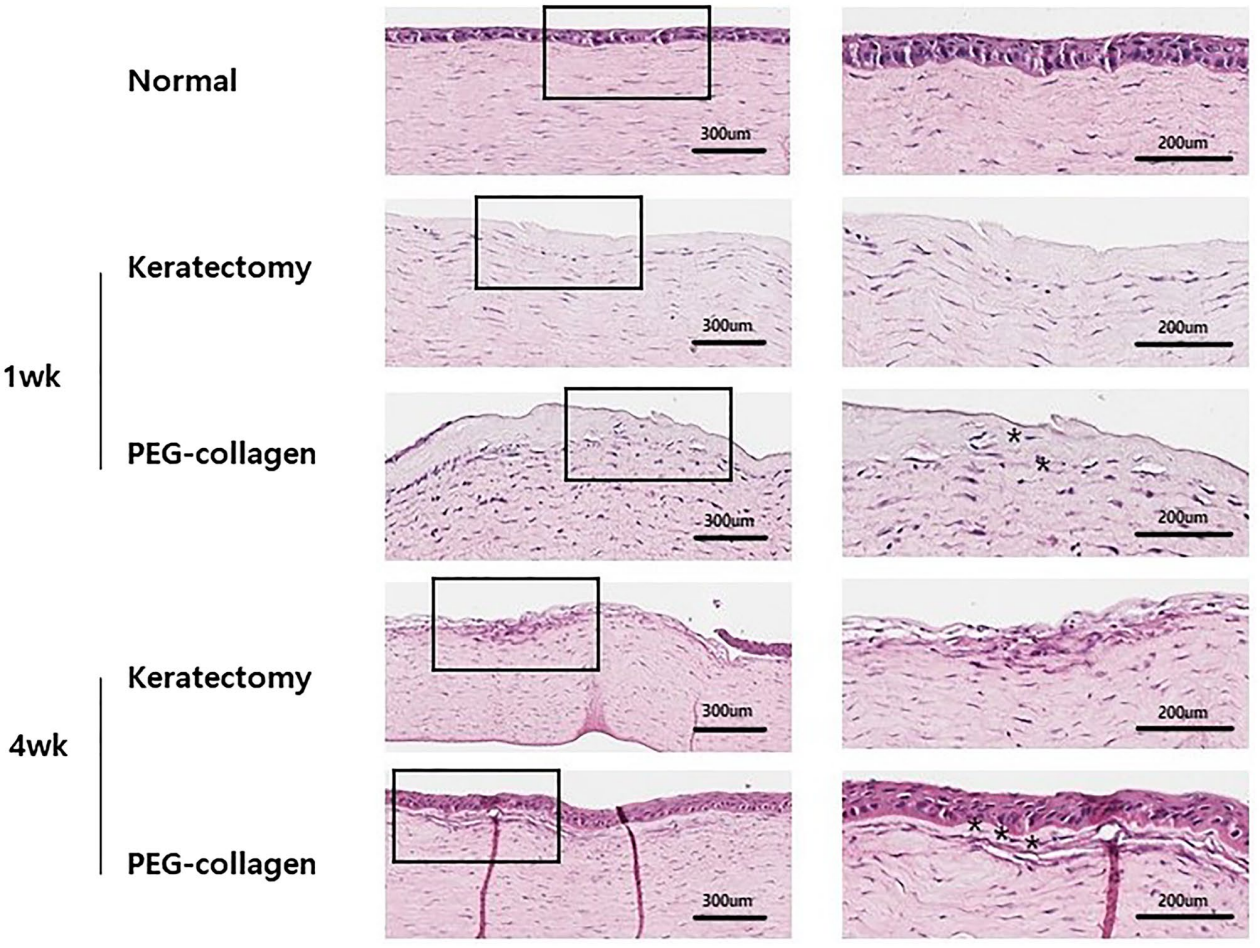
Light microscopy (hematoxylin and eosin staining) images of normal rabbit cornea and cornea subjected to keratectomy with or without polyethylene glycol (PEG)-collagen hydrogel application were obtained after 1 and 4 weeks of follow-up after surgery. The keratectomy-only group did not show complete epithelialization during the 4 weeks of follow-up, whereas the keratectomy with PEG-collagen hydrogel-treated group showed multilayered epithelial cell migration and proliferation. Cellular infiltrates were found in PEG-collagen hydrogel (*), but no significant inflammatory cell infiltration was noted.

**Figure 6:**
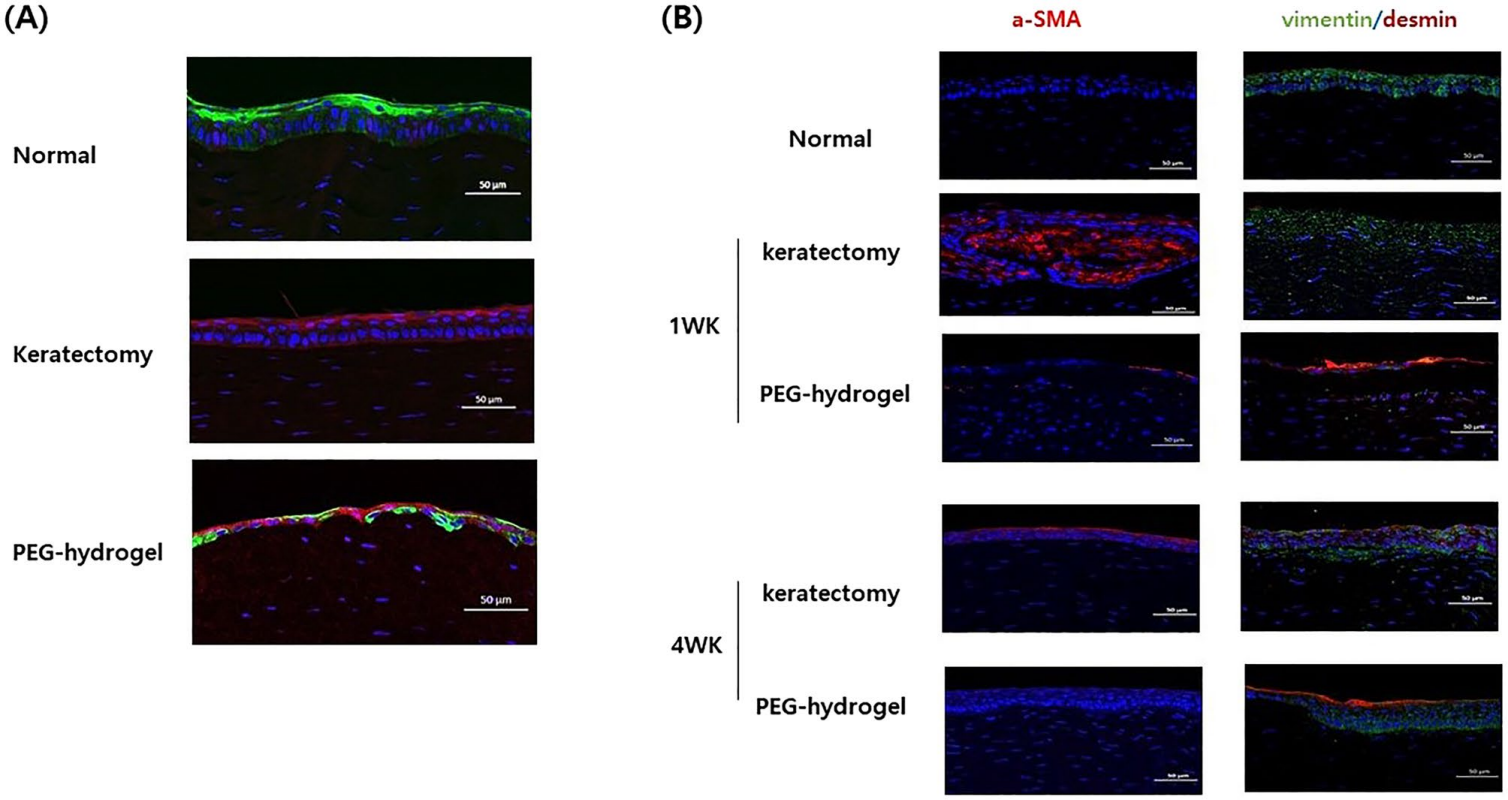
Fluorescent confocal microscopy images of rabbit keratectomy cornea without treatment and with polyethylene glycol (PEG)-collagen hydrogel application. (**A**) Cy5 stained with PEG-collagen hydrogel (magenta) was maintained underneath a multilayered, migrated epithelial layer stained with CK3 (green) after 1 week of keratectomy. (**B**) Immunohistochemical staining of α-smooth muscle actin (α-SMA), vimentin, and desmin markers for myofibroblasts in the keratectomy-subjected rabbit cornea over 1 week and 4 weeks. Note the high density of α-SMA (red) and vimentin (green) in the anterior stroma of rabbit corneas at 1 week after keratectomy alone. After 4 weeks, α-SMA (red) expression remained in the epithelium in the keratectomy-only group, whereas low α-SMA expression was found in the keratectomy with PEG-collagen hydrogel-treated group. Desmin (red) was expressed in the epithelium of the keratectomy with PEG-collagen hydrogel-treated group after 1 to 4 weeks.

In the keratectomy-only group, H&E-stained specimens showed an epithelial defect after 1 week of surgery, and fibrotic cells were stacked on the center of the cornea at 4 weeks following surgery, with the epithelial cells failing to migrate and proliferate from the periphery. In contrast, the cornea treated with PEG-collagen hydrogel maintained PEG-collagen at the stromal defect site after 1 week of surgery, and the epithelium formed a multilayer over the hydrogel after 4 weeks of surgery, and cellular infiltrates were seen in PEG-collagen hydrogel (Fig. 5). Immunofluorescence confocal imaging after 1 week also found PEG-collagen hydrogel underneath the migrated epithelium, suggesting that the injected PEG-collagen was able to support the reepithelialization (Fig. 6A).

Individual staining of α-SMA, which is a conventional marker for myofibroblast associated with corneal haze, was performed in each group, and the double staining of vimentin and desmin was performed to further analyze the corneal wound healing process after keratectomy with or without PEG-collagen hydrogel application. Strong expression of α*-*SMA (red) and vimentin (green) in the anterior stroma was detected in the keratectomy-only group after 1 week of surgery, and α-SMA remained on the epithelium and vimentin in the anterior stroma after 4 weeks of surgery (Fig. 6B).

## Discussion

The human cornea has two unique characteristics. First, the cornea forms the anterior barrier and protects the inner portion of the eye from the external environment. Second, the cornea is the single most powerful focusing element of the eye providing approximately two-thirds of the eye’s refractive power^19^. Thus, the following are highly prioritized when considering corneal alternatives: fair transparency with regular anterior surface, adequate strength to overcome intraocular and blinking pressure, and biocompatibility to improve integration^3,9^. Corneal stroma consists of highly organized collagen in arranged fibrils (collagen types I, V, and VI) with uniform spacing and alignment, which results in the transparent characteristic of the cornea^11^. Collagen is a natural polymer that is a major component of the ECM and has been used as a scaffold in clinical applications^10,12–18^.

The currently available biomaterials used as corneal adhesives cannot be used as stromal substitutes. For instance, the PEG-based materials ReSure (Ocular Therapeutix Inc., MA, USA) or OcuSeal (Beaver-Visitec International, MA, USA) cannot be used as filling biomaterials due to fast and uncontrollable polymerization, low adhesiveness, and lack of the mechanical properties required for regeneration^20,21^. Preclinical pilot study was demonstrated using human cadaveric donor cornea from ECM microparticles have been used; however, immunologic reactions may be problematic^22^. The newly introduced GelCORE, a gelatin-based adhesive, also showed in vivo biocompatibility and high tissue adhesion for 14 days^7^. A longer duration of observation is needed regarding zoonotic disease transfer or immunologic reactions, and the replacement of gelatin by host collagen should be monitored. Very recently, the performance of fully synthetic collagen-like peptide (CLP)-PEG-fibrinogen LiQD was equivalent to that of syngeneic grafts in vivo, suggesting a role as an alternative to corneal transplantation^6^. We chose a 4ARM PEG collagen formulation with 8% PEF due to its superior transparency both when cells are present and absent in vitro^18^. These PEG-based hydrogels were expected to be able to firmly adhere to the stromal bed due to the reactivity of the NHS moieties with primary amines in the host stromal collagen^17,18^.

Crosslinked collagens are used as hydrogel, sponge, or film and tailored to the shape of the defect site^17,23^. Recently, three-dimensional bioprinting has enabled the adjustment of the size and depth of the defect^24,25^. We used liquid in situ-forming hydrogel of collagen type I crosslinked via multifunctional PEG-NHS instead of solid alternative to treat corneal wounds^18,26^. Liquid hydrogels would have advantages over solid materials in terms of being easier to handle and having no need for being sutured or surgery. However, the controllable gelation time and initiation method, including the use of UV, visible light, or chemicals, need to be considered for purposes of safety and clinical approach. In addition, the overflow of liquid hydrogels from the site of the defect, if not carefully controlled, may prevent epithelial migration over the hydrogel^26^. Filling the defect would make it easy to individualize the various morphologies of corneal defects, and negating the irregular astigmatism by providing a smooth refracting surface is expected to improve vision even with the persistent corneal opacity of scarring^27^. In vitro, we found that the underlying letter was readable in both 0.4% and 0.8% of PEG-collagen hydrogel. However, corneal haze was found in both the keratectomy-only group and the PDG-collagen hydrogel-treated group, in vivo. Histological findings revealed that the PEG-collagen hydrogel, after its application into the corneal stromal defect site, was observed underneath a multilayered epithelial layer, which suggested that PEG-collagen hydrogel maintained in the stromal defect and epithelial migration was not inhibited by hydrogels. We further continued the in vivo observation and performed clinical assessments, including slit-lamp examination, IOP evaluation, AS-OCT, and other tests using Pentacam to analyze the biocompatibility of in situ-forming PEG-collagen hydrogels in vivo.

Corneal mechanical strength and structural integrity originate mainly from the collagen present in the stroma^22,28^. The mechanical properties of non-crosslinked collagen are difficult to control, which can pose a challenge. Therefore, we combined a natural polymer, collagen, and a synthetic polymer, PEG, to achieve adequate strength and control degradation^29^. A prominent advantage of PEG is its ability to control the physical and chemical properties in crosslinked networks^30^. Moreover, PEG is FDA-approved for use in clinical settings as an ocular sealant^20^. Fernandes-Cunha et al. have previously reported that collagen crosslinked with 3-arm PEG at various concentrations had a higher storage modulus than non-crosslinked collagen^18^. In the current study, we found that PEG-collagen hydrogel was maintained stably in the rabbit cornea up to 4 weeks after application. Our in vivo study showed that PEG-collagen hydrogel exhibited sufficient strength and elasticity to endure external (blinking) and internal (IOP) forces.

Results of the clinical analysis of AS-OCT revealed that the corneal thickness was restored to its normal range in the PEG-collagen-treated rabbit cornea, whereas this thickness decreased in the defective rabbit cornea without treatment. Thus, PEG-collagen hydrogel may play the role of temporary filler until the ECM is repaired. In photorefractive keratectomy, where the epithelium with underlying Bowman’s layer and anterior stroma is removed, stromal remodeling takes several months for completion^31^. During the wound healing process, aberrant ECM remodeling can lead to permanent corneal scar^31,32^. In a previous study in rabbits where superficial stroma was manually removed like in our study, it was shown that the wound completely epithelialized by day 7 and stomal remodeling continued until days 14–28; the findings were comparable to those of the human keratorefractive study^33^. We hypothesize that PEG-collagen hydrogel effectively and stably fills the stromal defect and enables rapid epithelial coverage at the beginning. This gain time for normal stromal wound healing process involves keratocyte, proinflammatory, and profibrotic cytokine production and ECM-degrading enzyme activity. A deeper stromal defect filled with PEG-collagen is warranted to examine the stromal remodeling including the donor keratocytes grow into the matrix and replace the donor collagen. We stained the intermediate filament markers—vimentin and desmin—and α-SMA to analyze the dynamics of their expression during myofibroblast differentiation after keratectomy with or without PEG-collagen hydrogel application. The normal cornea expresses epithelial α-SMA, vimentin, and desmin; however, anterior stromal staining of the markers has shown that they play a role in corneal scarring^34^. Detailed molecular studies investigating the effect of PEG-collagen hydrogel on the corneal wound healing process need to be conducted.

Clinical examination following injectable PEG-collagen filling at the stromal site showed no significant adverse effects, such as corneal infiltration, anterior chamber reaction, and hypersensitivity. The immune privilege of the cornea enables the implantation of various therapeutic apertures in the corneal stroma, such as the corneal inlay and intracorneal segment ring^35,36^. Due to the ubiquitous presence of PEG in cosmetic, cleaning, and personal care products, people can develop antibodies against the polymer^37^. These antibodies can provoke hypersensitivity reactions and accelerate biodegradation, decreasing the therapeutic efficacy of PEG^37^. There is a possibility that the PEG-collagen filling can also be used safely considering its marked association with the immune system. In the long term, further observations are needed to evaluate the effect of PEG-collagen on the absorption of disorganized collagen fibers and other matrix materials secreted by myofibroblasts. Limitation of this study is that we did not analyzed time dependent changes of underlying stroma and endothelium and overlying epithelium of the PEG-collagen, and only observed the 1 week and 4 weeks of results of the treated cornea. Moreover, the fate or role of treat PEG-collagen in the wound healing process is not clearly solved. Nevertheless, we have shown the clinical efficacy of the PEG-collagen hydrogel in vivo and found that the hydrogel filling at the defect site was stable and safe for 4 weeks after surgery. Although the PEG-collagen treated cornea did not show a perfectly smooth and transparent surface, the cornea curvature and thickness were tolerable and were maintained during the follow-up period. In our rabbit model, no steroids, antibiotics, or lubricant eyedrops were instilled after PEG-collagen injection. In the clinical setting, it may be possible for meticulous treatment during wound healing to restore transparency, and customized contact lenses or excimer laser surgery may lessen the astigmatism. Although further studies with longer duration are warranted, we demonstrated that the PEG-collagen hydrogel could be applied without major issues regarding cytotoxicity or foreign body reactions as a corneal stromal defect wound repair matrix.

## Materials and methods

### Materials

Unless otherwise noted, all chemicals and solvents were of analytical grade and used as advised by the manufacturer. Four-arm PEG succinimidyl glutarate was purchased from JenKem Technology (Texas, USA). Collagenase, dimethyl sulfoxide, fluorescamine, sodium hydroxide solution (1.0 N), bovine serum albumin, fibronectin, cholera subunit A, insulin, Triton-X, and Cell Counting Kit-8 reagent were purchased from Sigma-Aldrich (St. Louis, MO, USA). Phosphate-buffered saline (PBS; 10 × ; pH 7.4), Slide-A-Lyzer Dialysis Kit (3.5 kDa MWCO), collagen I bovine protein solution (5 mg/mL), collagenase, Dulbecco’s Modified Eagle Medium/Nutrient Mixture F-12 with 4-(2-hydroxyethyl)-1-piperazineethanesulfonic acid, insulin-transferrin-selenium, Dulbecco’s PBS, antibiotic–antimycotic, Live/Dead viability/cytotoxicity staining kit, and paraformaldehyde (PFA) were purchased from Thermo Fisher Scientific (Massachusetts, USA).

### Collagen crosslinking by 4-arm PEG-NHS

The pH of type I bovine collagen was neutralized using a solution of 1 M sodium hydroxide solution, deionized (DI) water, and 10 × PBS in a 3:57:20 ratio, as previously described^18^. Briefly, the collagen solution (5 mg/mL) was mixed with the neutralization solution in a 3:2 ratio, such that the final concentration of collagen was 3 mg/mL. Since the collagen crosslinking occurs with the primary amine group of lysine by NHS ester reaction, the number of lysine residues is important to determine the concentration of each chemical. Lysine constitutes 2.5–3.0% of collagen, amounting to a total of 75–100 lysine residues in the triple helix. Moreover, 3 mg of collagen corresponds to 7.5 × 10^−9^–7.5 × 10^−7^ mol of primary amine. The 4-arm PEG-NHS has four NHS groups per molecule; the molar equivalent amount of NHS groups is 1.875 mg per 3 mg of primary amine group in collagen. Thus, 18.75 µL of 100 mg/mL of 4-arm PEG-NHS provided the equivalent amount of NHS groups. Four-arm PEG-NHS was dissolved in PBS at a concentration of 100 mg/mL, and the solution was subsequently mixed with the neutralized collagen solution at the desired concentration (0.8% w/v) for 30 s at room temperature. Subsequently, the collagen gels were incubated for 10–15 min at 37 °C for the completion of gelation.

### Physical properties of PEG-collagen hydrogel

The mechanical properties of the collagen hydrogels were evaluated using an MCR 302 rheometer (Anton Paar, Germany). For the PEG-collagen hydrogels, different concentrations of 0.4 and 0.8% of 4-arm PEG-NHS solution was added to the neutralized collagen solution. Then the solution was mixed with a pipette. To ensure complete gelation, the resultant PEG-collagen hydrogels were incubated at 37 °C for 2 h, and then frequency sweeps from 0.1 to 10 Hz with a fixed 1% strain were performed.

### Animals and surgery

New Zealand White rabbits (male, 2.5–3.0 kg) were purchased from DooYeol Biotech. In total, 20 rabbits were subjected to keratectomy, and 10 rabbits were randomly assigned to each group (0.8% 4-arm PEG-collagen hydrogel PEG and keratectomy only). Animals were anaesthetized using an intramuscular injection of Zoletil™ 50 (15 mg/kg, Virbac, Carros, France) and xylazine hydrochloride (5 mg/kg, Bayer, Leverkusen, Germany). The right eye of each rabbit was chosen for surgery, and the contralateral eye served as the normal control. Three drops of alcaine (Alcon Laboratories, Inc., Ft. Worth, TX, USA) were applied to the cornea before surgery. Manual superficial stromal keratectomy was performed as previously described^38^. Briefly, a 5-mm-diameter keratectomy groove of consistent depth was created by a biopsy punch (Miltex, Inc. York, USA). Subsequently, a 27-gauge needle was used to inject air into the stroma to avoid corneal perforation, and the anterior stroma was manually dissected. Finally, using the freehand technique, approximately 20–30% of the anterior stroma was removed using a crescent blade. Following anterior lamellar dissection, the defect was filled with 0.8% 4-arm PEG-collagen-Alexa 647 hydrogels. After in situ-forming collagen hydrogel treatment, 3 min of drying time was allowed for complete gelation, and tarsorrhaphy was performed. Half of each group (N = 5 eyes), which was chosen randomly, was evaluated 1 week after surgery, while the other half was assessed 4 weeks postoperatively. One week and four weeks after the surgery, the animals were evaluated before being sacrificed. After euthanizing, the corneas were analyzed using light and confocal microscopy. All animal protocols were reviewed and approved by the Institutional Animal Care and Use Committee of the Catholic University of Korea (Institutional Review Board No: YEO-2019–015-01FA). This study was conducted in accordance with all applicable regulations and guidelines, including the ARRIVE 2.0 guidelines^39^.

### Clinical examination

Postoperative examinations were performed at 1 and 4 weeks after surgery. Slit-lamp biomicroscopy (Topcon Medical Systems Inc., USA) was performed to evaluate the anterior corneal surface and chamber, and images of the cornea were acquired using a digital camera (Sony Rx10M4, Japan) to compare its clarity and vascularity. Corneal clarity was graded manually from being completely transparent (0) to completely opaque (4) by the authors YHC, MJH, and KSN. Intraocular pressure (IOP) measurements were first performed using TonoVet (Helsinki, Finland). Because there was no calibration mode available for rabbits, a calibration mode for dogs was chosen, as previously introduced^40^. The central groove of the tonometer was kept in a horizontal position, and the distance was maintained at 4–8 mm between the tip of the probe and cornea. The measurement button was slightly pressed to enable the tip of the probe to hit the central cornea. IOP was measured before noon in each period to eliminate diurnal variations. Six consecutive measurements were recorded, and the resulting average IOP was reported.

Anterior segment optical coherence tomography (AS-OCT) (Visante OCT, Carl Zeiss Meditec, Inc., USA) was performed at week 4 postoperatively. Background illumination in the room was made constant by completely closing the door and minimizing room illumination. One anterior segment scan image centered over the pupil was acquired along the horizontal axis (between 0° and 180°). The scan direction was aligned until a full corneal reflex was achieved. Corneal topography and pachymetry were performed using a rotating Scheimpflug camera (Pentacam; Oculus, Wetzlar, Germany) 4 weeks postoperatively.

### Histology

After the completion of the study (on 1 and 4 weeks), tissues that required to be examined were isolated. The cornea was carefully separated from the eye without damaging it and washed using 1 × PBS. Tissues were fixed using 4% PFA solution overnight at 4 °C and then washed in 1 × PBS. The tissue segments were dehydrated in graded ethanol and embedded in paraffin, and 3–4 µm-thick sections were cut using a microtome. All the sections were subjected to hematoxylin and eosin (H&E) and immunofluorescence staining for histopathological analysis.

### H&E and immunofluorescence staining

For H&E staining, tissue Sects. (3–4 µm) were immersed in xylene (three times for 5 min); 100% (two times), 95%, 85%, and 70% ethanol for 1 min each; DI water for 1 min; and hematoxylin (BBC Biochemical) and eosin (BBC Biochemical) solutions for 10 to 20 s before washing with pure xylene.

For immunofluorescence staining, tissue slides were rehydrated by immersing in xylene (three times for 5 min); 100% (two times), 95%, 85%, and 70% ethanol for 5 min each; and DI water for 5 min. The antigen retrieval buffer (citrate buffer, pH 6.0) was preheated to 92–95 °C. Slides were immersed in the preheated solution for 5–10 min. The tissues were encircled with a hydrophobic barrier using a barrier pen. Tissues were further blocked with 2.5% Normal Horse Serum (Vector, Burlingame, CA, USA) for 30–60 min.

The slides were washed and incubated with primary antibodies: mouse monoclonal antibody against CK3 (ab68260, Abcam, Cambridge, UK) diluted 1:50, mouse monoclonal antibody against α-smooth muscle actin (α-SMA) (ab7817, Abcam, Cambridge, UK) diluted 1:50, anti-vimentin antibody (ab45939, Abcam, Cambridge, UK) diluted 1:100, and anti-desmin monoclonal antibody (MA1-2215, Invitrogen, Carlsbad, CA, USA) diluted 1:100 at 4 °C overnight. The next day, after washing with 1 × PBS (three times), the sections were incubated with goat anti-rabbit Alexa Fluor 488-conjugated secondary antibody (A-11008, Invitrogen, Carlsbad, CA, USA) or goat anti-mouse Alexa Fluor 546-conjugated secondary antibody (A-11003, Invitrogen, Carlsbad, CA, USA) at room temperature for 2 h in the dark. The slides were then mounted with a mounting medium containing 4′,6-diamidino-2-phenylindole (Vector). For negative controls, non-immune serum was used instead of the specific primary antibody. The corneal sections were observed and imaged with a confocal microscope (ZEISS LSM 880, Carl Zeiss AG, Oberkochen, Germany).

### Statistical analyses

All data are expressed as the mean ± standard deviation. Each experiment was performed at least three times, unless otherwise indicated. Statistical evaluation was performed using two-way analysis of variance. Results with *p* < 0.05 were considered statistically significant. Statistical analysis was performed using the statistical software GraphPad Prism 7.
